# Preferences for Personalized Text Message Appointment Reminders Among Outpatients in a Universal Health System: Cross-Sectional Study

**DOI:** 10.2196/81324

**Published:** 2026-04-07

**Authors:** Shih-Huai Hsiao, Yu-Chen Chuang, Shu-Ling Ma, Hui-Tzu Lin, I-Chen Lee, Pei-Shih Chen

**Affiliations:** 1 Tele-Healthcare Center, Kaohsiung Medical University Hospital, Kaohsiung Medical University, Kaohsiung, Taiwan; 2 Department of Public Health, College of Health Science, Kaohsiung Medical University, No. 100 Shih-Chuan 1st Road, Sammin District, Kaohsiung, 80756, Taiwan, 886-7-3121101 ext 6798; 3 Taiwan Health Insurance Association, Taipei, Taiwan; 4 Cardiovascular Division, Department of Internal Medicine, Kaohsiung Medical University Hospital, Kaohsiung Medical University, Kaohsiung, Taiwan; 5 Department of Nursing, Kaohsiung Medical University Hospital, Kaohsiung Medical University, Kaohsiung, Taiwan; 6 Clinical Skill Training Center, Department of Clinical Education and Skill Training, Kaohsiung Medical University Hospital, Kaohsiung Medical University, Kaohsiung, Taiwan; 7 Department of Healthcare Administration and Medical Informatics, College of Health Sciences, Kaohsiung Medical University, Kaohsiung, Taiwan

**Keywords:** SMS reminders, outpatient no-shows, patient preferences, behavioral design, health communication, personalized messaging, universal health coverage

## Abstract

**Background:**

SMS text messaging reminders are widely used to reduce missed outpatient appointments; however, evidence remains limited regarding which types of reminder content patients prefer, particularly within East Asian universal health systems. In Taiwan, minimal financial barriers to care and unrestricted access to secondary and tertiary hospitals contribute to high outpatient visit volumes and persistent no-show rates. These contextual features underscore the need for behaviorally informed and demographically tailored reminder strategies rather than uniform messaging approaches.

**Objective:**

This study aimed to examine patient preferences for 6 theory-guided SMS appointment reminder types and to identify the predictors of reminder preference related to demographic characteristics and health care utilization, with the goal of informing personalized reminder design for a forthcoming randomized controlled trial.

**Methods:**

We conducted a cross-sectional online survey among adults in Taiwan with prior outpatient experience. Six SMS reminder prototypes were developed based on behavioral communication principles and validated by a multidisciplinary expert panel using item-level content validity indices. Participants selected their preferred SMS reminder type and reported sociodemographic characteristics and recent health care utilization. Bivariate associations were examined using chi-square tests and one-way ANOVA, with Benjamini-Hochberg false discovery rate correction applied to control for multiple testing. To identify independent predictors of SMS reminder preference while adjusting for potential confounding, we fitted a multinomial logistic regression model with all covariates entered simultaneously.

**Results:**

A total of 1095 respondents completed the survey. General reminders and messages referencing prior missed appointments were most frequently preferred, whereas empathy-based or relationally framed messages were selected less often. In false discovery rate–adjusted univariate analyses, both age and sex were associated with SMS reminder preference. However, in the fully adjusted multinomial logistic regression model, age emerged as the only statistically significant independent predictor. Participants younger than 50 years were significantly more likely to prefer alternative reminder message types compared with the general reminder (adjusted odds ratio 1.64, 95% CI 1.18‐2.28; *P*=.003). Sex did not retain statistical significance after multivariable adjustment. Other sociodemographic characteristics and health care utilization variables, including education level, employment status, residential region, outpatient visit frequency, and recent missed appointments history, were not independently associated with reminder preference.

**Conclusions:**

Preferences for outpatient SMS reminder content vary systematically, with age representing the most robust independent predictor. Across the sample, concise and behavior-focused reminders were preferred over empathy-oriented or relational formats. These findings support age-informed tailoring of SMS reminder content and provide content-validated SMS prototypes for use in subsequent interventional research. The results offer formative evidence to guide the design of randomized trials aimed at reducing outpatient no-shows and improving the efficiency of ambulatory care delivery in Taiwan’s universal health care system.

## Introduction

Timely attendance at outpatient appointments is fundamental to maintaining care continuity, optimizing health care resource utilization, and achieving favorable clinical outcomes [1]. However, missed appointments (MAs), commonly referred to as “no-shows,” remain a persistent global challenge, with no-show rates in outpatient settings averaging approximately 23% [2]. These disruptions not only compromise the efficiency of health care delivery but also contribute to inequities in access and outcomes, particularly among patients facing socioeconomic disadvantages, transportation barriers, or prolonged wait times in specialty care services, such as cardiology and dermatology [3-6].

To address this issue, SMS reminders have been widely implemented and demonstrated to be effective in reducing no-show rates across various clinical contexts, including gastrointestinal procedures [7-9] and surgeries requiring anesthesia [10]. Compared with traditional telephone calls, which are labor-intensive and less scalable, SMS reminders offer a cost-effective and scalable solution for promoting appointment adherence [11]. Moreover, behaviorally informed message designs, such as emotionally salient language, appeals to social responsibility, or moral framing, have been shown to enhance patient engagement and accountability [7,12-14]. The inclusion of personalized content, such as references to prior MAs, has also been associated with further reductions in no-show rates [10,15,16].

Despite the growing body of evidence supporting the efficacy of SMS reminders, limited research has examined the optimal design of message content. Specifically, there is a lack of systematic investigation into which core components should be included, how linguistic and emotional framing influences patient behavior, and to what extent content should be tailored to individual characteristics. These characteristics may include insurance type, age, occupation, socioeconomic status, prior attendance history, disease severity, or the need for chronic follow-up care. Personalization strategies of this nature are likely critical to maximizing the impact of reminder interventions but have been insufficiently explored in the literature.

This gap is particularly salient in East Asian health care systems, such as Taiwan’s, where universal health coverage and unrestricted access to secondary and tertiary care may lead to distinct patterns of health care utilization. Under Taiwan’s National Health Insurance program, patients can access higher-level hospitals directly without referral and with minimal out-of-pocket expense, often resulting in frequent outpatient visits for relatively minor conditions [17]. The ease of access and low perceived urgency of such visits may contribute to elevated no-show rates; one tertiary medical center in Taiwan reported a no-show rate as high as 16.8% [18]. These contextual factors highlight the need for more effective and targeted reminder strategies that are responsive to patient behavior and health care system dynamics.

Specifically, the study aimed to (1) assess whether there was significant heterogeneity in preferences for various types of SMS content and (2) examine the extent to which these preferences were associated with patients’ sociodemographic characteristics, socioeconomic status, and recent health care utilization patterns.

## Methods

### Study Design and Participant Recruitment

This cross-sectional study used a nonprobability convenience sampling method. Participants were recruited through online platforms (eg, Facebook, LINE) and professional networks, including outreach by health care professionals.

Participants were eligible if they were adults aged 20 years or older, resided in Taiwan, had experience using SMS-capable mobile phones, and completed the online questionnaire with informed consent. Individuals were excluded if they lacked SMS access, had known cognitive impairments, or reported working in health care–related professions (eg, hospitals or clinics) to minimize potential response bias. The structured questionnaire was distributed electronically, and the data were collected anonymously over a 14-day period from February 25 to March 10, 2025. A total of 1152 responses were received, of which 1095 were valid, yielding a valid response rate of 95.05%.

### Instrument Development and Content Validity

#### SMS Reminder Messages Development

Grounded in behavioral theory and prior empirical research, this study developed 6 SMS appointment reminder prototypes that incorporate cognitive, emotional, and social cues to enhance patient relevance and behavioral efficacy. Message design was guided by the SMS content framework proposed by Acharya et al [19], with 4 core principles applied: the use of plain language, a neutral tone, explicit appointment details, and clear sender identification. Three principles, namely the inclusion of external links, preparation instructions, and emphasis on the consequences of nonattendance, were considered less applicable to general outpatient contexts. While the prototypes largely conformed to established design standards, contextual adaptation remains essential when applying screening-focused frameworks to broader clinical settings. The 6 SMS message types are summarized in Table 1 and are described as follows:

1. General reminder: a neutral notification containing appointment details [9-11,20].
2. Prior MA: referencing the patient’s history of missed visits and institutional policies. This type can further reduce the no-show rate by approximately 1% compared to standard reminders [14] and promote behavioral change through increased awareness of institutional norms [10].
3. Provider-focused empathy: highlighting the emotional burden and workload of health care staff, aiming to evoke moral responsibility and cooperation, especially in high-burden clinical settings [12].
4. Patient-focused empathy: emphasizing how MA may limit others’ access to care, particularly under Taiwan’s hospital-specific global budgeting system, where physician time and service volume are constrained. Such awareness may foster social responsibility and encourage patients to proactively cancel or reschedule. Empathy-based reminders have also been shown to reduce provider-patient tension [21].
5. Physician follow-up: simulating concern from the attending physician by referencing the patient’s upcoming visit and inviting them to return on the scheduled date. This tone, regardless of prior clinical contact, may enhance engagement and continuity of care, especially for patients with chronic conditions or long-term follow-up needs [22].
6. Supportive reminder: using friendly, conversational language to reduce psychological barriers and ease anxiety related to clinic visits. Warm, supportive messaging may improve follow-up motivation and engagement, particularly for patients who perceive medical visits as stressful [14].

The 6 SMS messages in their original Chinese form have been included in Multimedia Appendix 1.

**Table 1.** Theory-guided SMS reminder types with message content and technical specifications for outpatient appointment adherence.

Type of SMS reminder	Wording of SMS content in this study
General reminders	Hi! You have an appt. with [Dr Name] on the [Morning/Afternoon/Evening] of [Date]. To cancel or reschedule, please visit [clinic or hospital] booking system. We care about your health!
Prior missed appointment behavior	Hi! You have an appt. with [Dr Name] on the [Morning/Afternoon/Evening] of [Date]. Your Appt. No. is [Appt. No.]. If this MA results in a total of three no-shows without prior cancellation, you will no longer be eligible for remote booking. Future appt. must be made in person at the [clinic or hospital]. To cancel or change, please visit [clinic or hospital] booking system. We care about your health!
Joint empathy communication type 1: provider-focused	Hi! You have an appt. with [Dr Name] on the [Morning/Afternoon/Evening] of [Date]. Your Appt. No. is [Appt. No.]. If you don’t show up and don’t cancel, it may cause trouble for medical staff. To cancel or reschedule, please visit [clinic or hospital] booking system. We care about your health!
Joint empathy communication type 2: patient-focused	Hi! You have an appt. with [Dr Name] on the [Morning/Afternoon/Evening] of [Date]. Your Appt. No. is [Number]. If you miss your appt. without cancellation, it may affect other patients’ access to care. To cancel or reschedule, please visit [clinic or hospital booking system]. We care about your health!
Gentle and friendly (physician follow-up)	Hi! [Dr Name] cares about you. You have a [specialty] appt. on the [Morning/Afternoon/Evening] of [Date]. To cancel or reschedule, please visit [clinic or hospital] booking system. We care about your health!
Supportive reminder	Hope you’re doing well! [Dr Name] cares about your health. You have a [specialty] appt. on the [Morning/Afternoon/Evening] of [Date]. Your Appt. No. is [number]. To cancel or reschedule, please visit [clinic or hospital] booking system. We care about your health!

#### Content Validity Examination of SMS Message

To evaluate the content validity of the SMS messages, a multidisciplinary panel of 12 experts was convened. The panel included 2 clinicians, 3 managers from patient registration and cashier services, 2 language educators, 3 nursing supervisors responsible for outpatient care, and 2 professionals from insurance and customer service departments. All panel members had prior personal experience as patients or caregivers and demonstrated strong communication competencies, ensuring a broad range of perspectives from clinical, administrative, linguistic, and consumer viewpoints. No significant demographic differences were found among the expert subgroups (*P*>.05).

Each SMS prototype was assessed using a 5-point Likert scale across 3 domains: semantic clarity, content relevance, and ease of comprehension. The item-level content validity index for all 6 messages exceeded the standard threshold of 0.78 [17,23], confirming adequate content validity.

### Statistical Analysis

Descriptive statistics summarized participant characteristics. Chi-square tests and one-way ANOVA were used to examine bivariate associations between participant characteristics and SMS reminder preferences. To control for inflated type 1 error due to multiple comparisons, the Benjamini-Hochberg false discovery rate (FDR) procedure was applied to the bivariate tests [24].

To adjust for potential confounding and identify independent predictors of SMS reminder preference, we fitted a multinomial logistic regression model with SMS reminder preference as the outcome. The general reminder SMS was specified as the reference category. Demographic characteristics and health care utilization variables were entered simultaneously as covariates, and the results are reported as adjusted odds ratios with 95% CI. Statistical significance was set at *P*<.05 (2-tailed). All analyses were conducted using SPSS (version 29.0.1.0).

### Ethical Considerations

This study was reviewed and approved by the Institutional Review Board of Kaohsiung Medical University Chung-Ho Memorial Hospital (IRB number: KMUHIRB-E(I)-20240047; approval date: February 3, 2025). Electronic informed consent was obtained from all participants prior to survey submission. Participation was voluntary, anonymity was ensured, and no identifiable personal information was collected. All study procedures adhered to institutional ethical guidelines and the principles of the Declaration of Helsinki.

## Results

### Participant Characteristics

#### Demographic and Socioeconomic Status

Among the 1095 participants, female participants predominated (n=851, 77.7%). The mean age was 43.2 years (SD 12.37), with 65.8% (n=720) aged below 50 years. Most (n=726, 66.3%) respondents held a university or college degree, followed by postgraduate qualifications (n=272, 24.8%), and secondary-level education or below (n=97, 8.9%).

The largest occupational groups were professionals (n=352, 32.1%) and clerical and administrative staff (n=254, 23.2%), with smaller proportions in sales or service roles, technical support, senior management, and unemployed categories. Nearly half (n=515, 47%) resided in the Kaohsiung-Pingtung region, and the majority (n=800, 73.1%) lived with others. A minority (n=216, 19.7%) were unmarried or living alone (n=79, 7.2%; Table 2).

**Table 2.** Demographic and socioeconomic distribution of the respondents.

Variable	Respondents
Sex, n (%)	
Male	244 (22.3)
Female	851 (77.7)
Age (y), mean (SD)	43.2 (12.38)
Age group (y), n (%)	1095 (100)
<50	720 (65.8)
≥50	375 (34.2)
Education level, n (%)	
Senior high school or lower education	97 (8.9)
College or university education	726 (66.3)
Postgraduate degree	272 (24.8)
Occupation category, n (%)	
Senior executives and decision-makers	114 (10.4)
Professionals	352 (32.1)
Technicians and assistants	96 (8.8)
Clerical and administrative staff	254 (23.2)
Sales and service workers	147 (13.4)
Other occupations	54 (4.9)
Unemployed	78 (7.1)
Employment status, n (%)	
Retired	109 (10)
Employed	897 (81.9)
Unemployed	89 (8.1)
Region of residence, n (%)	
Kaohsiung-Pingtung region	515 (47)
Outside Kaohsiung-Pingtung region	580 (53)
Living arrangement, n (%)	
Unmarried	216 (19.7)
Living alone	79 (7.2)
Living with others	800 (73.1)
Outpatient visits in the past 3 months, n (%)	
0 visit	192 (17.5)
1‐3 visits	658 (60.1)
4 or more visits	245 (22.4)
Missed outpatient appointment in the past 3 months, n (%)
No missed appointment	923 (84.3)
Missed appointment at least once	172 (15.7)
Frequency of missed appointments in the past 3 months, n (%)
Fewer than 3 times	1074 (98.1)
Three times or more	21 (1.9)

#### Health Care Utilization

In the preceding 3 months, 60.1% (n=658) of the participants had 1‐3 outpatient visits, 22.4% (n=245) reported 4 or more visits, and 17.5% (n=192) had not accessed outpatient services. Additionally, 15.7% (n=172) reported at least 1 MA, while 84.3% (n=923) had no such record.

Of note, 17.5% (n=192) of the participants reported no outpatient visits in the past 3 months, yet 15.7% (n=172) indicated at least 1 MA. This discrepancy may reflect recall bias or differences in participants’ interpretation of MAs, such as counting canceled or forgotten appointments that were never formally registered as visits.

### Distribution of SMS Reminder Preferences

As shown in Table 3, nearly half (n=520, 47.5%) of the respondents preferred reminders referencing “Prior MAs” behavior, followed by general reminders (n=341, 31.1%). Fewer participants selected messages with an empathetic tone, including provider-focused empathy (n=48, 4.4%) and patient-focused empathy (n=60, 5.5%). Relational reminders, such as physician follow-up (n=53, 4.8%) and supportive reminders (n=73, 6.7%), accounted for a combined total of 11.5% of the preferences. These results indicate a predominant preference for practical and behavior-linked reminder formats over affective or relational messaging.

Because 4 SMS categories (provider-focused empathy, patient-focused empathy, physician follow-up, and supportive reminders) were selected by relatively few participants, the corresponding subgroup cell sizes in the chi-square analyses were small. Therefore, statistical comparisons involving these categories should be interpreted with caution.

Male respondents showed relatively higher proportions selecting supportive or relational message types, although these differences were not statistically significant after FDR correction.

**Table 3.** Distribution of preferred SMS reminder types (N=1095).

SMS reminder type	Values, n (%)
Prior missed appointment reminder	520 (47.5)
General reminder	341 (31.1)
Supportive reminder	73 (6.7)
Patient-focused empathy	60 (5.5)
Physician follow-up	53 (4.8)
Provider-focused empathy	48 (4.4)
Total	1095 (100)

### Associations Between Demographic, Socioeconomic, and Health Care Utilization Characteristics and Preferred SMS Reminder Type

Analyses of demographic and health care utilization characteristics showed several initial associations with SMS reminder type preferences. As summarized in the cross-tabulations, gender, age group, education level, occupation, residential region, and living arrangement each demonstrated significant differences across message categories in unadjusted chi-square and ANOVA analyses (Table 4). For example, women and younger adults more frequently selected general or prior missed appointment reminders, whereas older adults showed comparatively higher proportions selecting empathetic or supportive formats. Occupational variation was also observed, with clerical and professional workers tending toward standard reminders, while senior executives reported a greater share of emotionally supportive preferences. These subgroup differences, however, did not persist following correction for multiple comparisons.

Health care utilization variables exhibited similar patterns. Participants with 1 to 3 outpatient visits in the past 3 months tended to prefer neutral, task-oriented reminders, while those with at least 1 recent MA were more likely to choose messages explicitly referencing prior absences (Table 5).

**Table 4.** Associations between demographic characteristics and preferred SMS reminder type.

Variable	General reminder, n (%)	Prior missed appointment behavior, n (%)	Provider-focused empathy, n (%)	Patient-focused empathy, n (%)	Physician follow-up, n (%)	Supportive reminder, n (%)	Chi-square or *F* test (*df*)	*P* value
Sex							26.554^a^ (5)	.001
Male	67 (19.6)	102 (19.6)	12 (25.0)	21 (35.0)	11 (20.8)	31 (42.5)		
Female	274 (80.4)	418 (80.4)	36 (75.0)	39 (65.0)	42 (79.2)	42 (57.5)		
Age (y)								
Mean age (SD)	45.82 (12.43)	40.05 (12.33)	43.13 (12.41)	45.62 (11.93)	50.45 (11.19)	45.53 (13.39)	19.54^b^	.001
<50, n (%)	197 (57.8)	395 (76.0)	30 (62.5)	32 (53.3)	24 (45.3)	42 (57.5)		
≥50, n (%)	144 (42.2)	125 (24.0)	18 (37.5)	28 (46.7)	29 (54.7)	31 (42.5)		
Education								
Senior high or less	28 (8.2)	46 (8.8)	3 (6.3)	8 (13.3)	8 (15.1)	8 (11.0)	5.977^b^ (10)	.82
College or university	225 (66.0)	356 (68.5)	31 (64.6)	35 (58.3)	35 (66.0)	44 (60.3)		
Postgraduate	88 (25.8)	118 (22.7)	14 (29.2)	17 (28.3)	14 (26.4)	21 (28.8)		

a Chi-square test.

b *F* test.

**Table 5.** Associations between health care utilization characteristics and preferred SMS reminder type.

Variable	General reminder, n (%)	Prior MA^a^ behavior, n (%)	Provider-focused empathy, n (%)	Patient-focused empathy, n (%)	Physician follow-up, n (%)	Supportive reminder, n (%)	Chi-square (*df*)	*P* value
Outpatient visits (past 3 months)							5.112 (10)	.88
0 visits	56 (5.1)	89 (8.1)	10 (9.4)	12 (11.0)	11 (10.4)	14 (13.3)		
1‐3 visits	201 (58.9)	315 (60.6)	31 (64.6)	37 (61.7)	33 (62.3)	41 (56.2)		
≥4 visits	84 (24.6)	116 (22.3)	7 (14.6)	11 (18.3)	9 (17.0)	18 (24.7)		
MAs (past 3 months)							3.267 (5)	.66
None	286 (83.9)	446 (85.8)	41 (85.4)	50 (83.3)	44 (83.0)	57 (78.1)		
≥1 MA	56 (16.4)	74 (14.2)	7 (14.6)	10 (16.7)	9 (17.0)	16 (21.9)		
Frequency of MAs							10.464 (5)	.06
<3 MAs	331 (97.1)	490 (94.2)	47 (97.9)	59 (98.3)	52 (98.1)	69 (94.5)		
≥3 MAs	10 (2.9)	4 (0.8)	1 (2.1)	1 (1.7)	1 (1.9)	4 (5.5)		

a MA: missed appointment.

To address the potential inflation of type 1 error due to multiple comparisons, the Benjamini-Hochberg FDR correction was applied across all demographic and utilization variables. After adjustment, only age and gender remained statistically significant predictors of SMS preference. All other variables, including education, occupation, region, living arrangement, outpatient visit frequency, and missed appointment history, no longer met the adjusted significance threshold. Multinomial logistic regression yielded consistent results, confirming age and sex as the only independent predictors after controlling for all covariates.

Although initial chi-square and ANOVA results suggested that several demographic and health care utilization variables were associated with SMS reminder preferences, these patterns did not remain statistically stable after controlling for multiple comparisons. After applying the Benjamini-Hochberg FDR correction, only age and sex remained significant predictors, whereas education level, occupation, residential region, living arrangement, outpatient visit frequency, and missed appointment history were no longer statistically significant (Table 6).

**Table 6.** Summary of false discovery rate (FDR)–corrected significance across all demographic and health care utilization variables.

Variable	Chi-square and *F* test (*df*)	Original *P* value	FDR-adjusted *P* value	Significant after FDR?
Sex	26.554 (df)^a^	.001	.006	Yes
Age (y; continuous)	19.54 (df)^b^	.001	.003	Yes
Education	5.977 (df) ^a^	.82	.98	No
Outpatient visits (past 3 months)	5.112 (df)^a^	.88	.88	No
Missed appointments (past 3 months)	3.267 (df)^a^	.66	.99	No
Frequency of missed appointments	10.464 (df)^a^	.06	.13	No

a Chi-square.

b *F* test.

To identify independent predictors of SMS reminder preference while simultaneously adjusting for all covariates, a multinomial logistic regression model was constructed. The full results of this multivariable analysis are presented in Table 7, including adjusted odds ratios (aORs), 95% CI, and *P* values.

As shown in Table 7, the multinomial logistic regression analysis identified age as the only statistically significant independent predictor of SMS reminder preference after simultaneous adjustment for all covariates. Participants younger than 50 years were significantly more likely to prefer alternative reminder message types compared with the general reminder SMS (aOR 1.64, 95% CI 1.18‐2.28; *P*=.003). In contrast, sex did not remain independently associated with reminder preference after multivariable adjustment (aOR 1.23, 95% CI 0.87‐1.73; *P*=.24). Other sociodemographic characteristics and health care utilization variables, including education level, employment status, residential region, living arrangement, outpatient visit frequency, and recent MAs history, were not statistically significant in the fully adjusted model.

**Table 7.** Multinomial logistic regression analysis of factors associated with SMS reminder preference^a^.

Predictor and category	aOR^b^	95% CI	*P* value
Sex			
Male (vs female)	1.23	0.87-1.73	.24
Age group			
<50 years (vs ≥50 years)	1.64	1.18-2.28	.003
Education level			
Less than or equal to high school (vs graduate+)	1.58	0.90-2.78	.11
College (vs graduate+)	1.12	0.81-1.55	.50
Employment status			
Retired (vs unemployed)	1.33	0.64-2.78	.44
Employed (vs unemployed)	1.65	0.90-3.01	.11
Residential region			
Kaoping region (vs non-Kaoping)	0.79	0.60-1.03	.08
Living arrangement			
Unmarried (vs with others)	1.31	0.91-1.89	.15
Living alone (vs with others)	1.21	0.72-2.06	.47
Outpatient visits (past 3 months)			
0 visits (vs ≥4)	1.19	0.78-1.81	.42
1-3 visits (vs ≥4)	1.15	0.84-1.58	.40
Missed appointments (past 3 months)			
≤3 times (vs ≥3)	2.08	0.86-5.04	.10

a Outcome variable: preference for SMS reminder type. Reference category: general reminder SMS. Model: multinomial logistic regression (all covariates entered simultaneously).

b aOR: adjusted odds ratio.

## Discussion

### Principal Findings

The primary objective of this study was to examine public preferences regarding SMS reminder content in preparation for a forthcoming large-scale randomized controlled trial. In the planned randomized controlled trial, participants will be randomly allocated to either an intervention group, which will receive a standardized SMS reminder seven days prior to their scheduled appointment containing details such as the appointment date, time, department, and queue number, or a control group that receives no reminder. Insights from this study will inform the development of effective SMS content strategies to enhance outpatient appointment adherence. The following sections address several key issues that merit further discussion.

### Sample Representativeness

The observed preference for concise, behaviorally framed SMS reminders, particularly the general reminder and prior MA formats, was most prominent among women, younger adults, and highly educated respondents. These patterns align with the demographic profile of the study sample, in which the majority were female (n=851, 77.7%), under age 50 (n=720, 65.8%), and held a college degree or above (91.1%).

Prior literature indicates that women are more likely than men to assume caregiving responsibilities within households, maintain greater engagement with preventive health services, and demonstrate higher willingness to participate in self-administered online questionnaires related to health behaviors and health care use [25-27]. These factors may contribute to their increased representation in digital health surveys.

Furthermore, women consistently report higher health literacy and mobile engagement for health-related purposes, including appointment scheduling, medication reminders, and symptom monitoring, compared with men of the same age groups [28]. Studies have also shown that women exhibit stronger preferences for receiving health information via SMS or other asynchronous communication channels, which may further elevate their likelihood of responding to an online survey focused on SMS reminder content. In contrast, men tend to participate at lower rates in health communication surveys unless participation is tied to an active medical condition or clinical follow-up requirement [29].

Collectively, the predominance of women in this sample likely reflects well-documented patterns in digital health engagement rather than a study-specific recruitment artifact. Nevertheless, the skewed sex distribution requires the cautious interpretation of aggregated preference patterns. To address this concern, sex was explicitly incorporated as a key covariate in the multinomial logistic regression analysis. After multivariable adjustment, sex did not remain independently associated with SMS reminder preference, indicating that the observed preference patterns were not solely driven by sample composition.

### Potential Data Inconsistency in Appointment and Missed Visit Reporting

A minor discrepancy was observed between outpatient visit reports and MA data. Specifically, 17.5% (n=192) of the respondents reported no outpatient visits in the past 3 months, but 15.7% (n=172) reported at least 1 MA. This inconsistency may reflect recall bias or variable interpretations of MAs, such as canceled or forgotten visits not formally captured by hospital systems. Our findings echo those of de Reuver and Bouwman [30], who observed that type 1 and type 2 reporting errors are common in self-reported utilization data.

### Preference Heterogeneity

The chi-square analyses indicated that participants most frequently selected general reminders and prior MA messages, while empathy-oriented or relationally framed messages were selected far less often. This suggests that patients prioritize clarity and actionable information over affective framing when receiving appointment-related communication. These findings are consistent with Hallsworth et al [16], who demonstrated that reminders containing cost-related information and clear behavioral cues were more effective than emotionally framed messages.

### Preferences Differ by Sociodemographic and Health Care Utilization Characteristics

The results demonstrated meaningful variation in SMS reminder preferences by age and sex, whereas other demographic and behavioral characteristics did not show stable associations after correction for multiple testing.

After correction for multiple testing, age and sex remained statistically significant in FDR-adjusted univariate analyses. However, when all covariates were entered simultaneously into the multinomial logistic regression model, age emerged as the only independent predictor of SMS reminder preference. Sex did not retain statistical significance after multivariable adjustment.

After multivariable adjustment, age emerged as the only independent predictor of SMS reminder preference, whereas sex did not retain statistical significance. Other variables, including education, occupation, residential region, living arrangement, outpatient visit frequency, and recent MAs, lost statistical significance following the FDR adjustment.

Descriptively, men demonstrated a more even distribution of preferences across message types; however, these differences did not remain statistically significant after multivariable adjustment and should be interpreted cautiously.

These findings indicate that designing personalized SMS strategies based primarily on age and sex may be more appropriate than tailoring messages by education level or utilization history. This pattern is consistent with Crutchfield et al [31] and Mohammed Selim et al [32], who observed that sociodemographic characteristics influence responsiveness to reminder messages. An Israeli randomized controlled trial further found that older adults and those with prior MAs were more responsive to emotionally framed messages [33]. Junod Perron et al [34], however, emphasized that communication mode (SMS vs telephone) may influence engagement differently than message framing.

Because multiple chi-square tests were conducted, the Benjamini-Hochberg FDR procedure was applied to reduce the risk of type 1 error. After correction, only age and sex remained statistically significant predictors of SMS preference. Residential region, living arrangement, and all health care utilization variables, including outpatient visit frequency and recent MAs, were not significant after correction.

These results were corroborated by the multinomial logistic regression model, in which age and sex remained the only independent predictors after mutual adjustment. Overall, these findings indicate that although unadjusted analyses suggested broader subgroup differences, only age- and sex-based differences demonstrated sufficient stability to warrant consideration in tailored SMS design.

It should be noted that several message types, particularly empathy-based formats, had limited subgroup counts. The observed associations for these categories may therefore lack statistical robustness and should be interpreted conservatively. The revised text avoids overstating preference differences in low-frequency groups.

### Content Validity Assessment

To ensure the appropriateness and clarity of SMS content prior to trial implementation, this study used item-level content validity indices based on 9 communication design principles derived from existing literature [17,19,23]. A multidisciplinary expert panel of 12 evaluators assessed the messages, exceeding recommended minimum panel sizes for content validation.

Although the panel did not include formal patient representatives, all members had extensive experience interacting with patients in outpatient or hospital settings. These perspectives provided practical insights grounded in real-world communication processes. This limitation is acknowledged, yet expert-based content validation remains standard practice in early formative research.

### Rationale for Age Cutoff at 50 Years

The decision to dichotomize age at 50 years was justified by both statistical and contextual considerations. Statistically, this threshold yielded significant differences in SMS preferences. Contextually, adults around age 50 years in Taiwan frequently assume dual caregiving responsibilities for both aging parents and children, which may influence their engagement with health care communication [35].

### Limitations

This study relied on convenience sampling through an online survey, which may limit representativeness. The predominance of female respondents reflects a well-documented pattern in online and digital health surveys, in which women are more likely than men to participate, particularly in studies related to health behaviors and health care communication [36,37]. To address this imbalance, sex was explicitly incorporated as a covariate in both univariate analyses and the multinomial logistic regression model. After multivariable adjustment, sex did not remain independently associated with SMS reminder preference, suggesting that the observed patterns were not solely driven by sample composition. Nevertheless, absolute preference proportions should be interpreted with caution, particularly for male patients. Future studies may enhance representativeness through targeted recruitment strategies and the inclusion of more inclusive gender response options, in line with emerging best practices in survey methodology [38].

### Conclusion

This study demonstrates that preferences for SMS appointment reminder content vary systematically among outpatients, with age emerging as the most robust independent predictor. General reminders and prior missed appointment messages were most consistently preferred, whereas empathy-based or relational formats were selected far less frequently. After applying FDA correction and conducting multinomial logistic regression, age remained the only stable independent predictor of SMS reminder preference, whereas sex did not retain statistical significance after multivariable adjustment. Beyond predictor effects, the observed preference for concise, action-oriented messages underscores the potential importance of clarity and behavioral specificity when designing SMS reminders in Taiwan’s universal health coverage context. Content validity assessment further confirmed that the 6 SMS prototypes possess adequate semantic clarity and cultural appropriateness for use in subsequent interventional research.

Although convenience sampling limits generalizability, the results offer valuable formative evidence to guide the development of SMS interventions aimed at improving appointment adherence. Future randomized controlled trials should evaluate the real-world effectiveness of these demographically tailored and behaviorally informed reminder formats in reducing outpatient no-show rates and supporting more efficient use of ambulatory care resources.

## Supplementary material

10.2196/81324 Multimedia Appendix 1 The original 6 prototypes of SMS reminders (Chinese and English).

## Abbreviations

aOR: adjusted odds ratio
FDR: false discovery rate
MA: missed appointment
