# Supplementary material for: Preferences for Personalized Text Message Appointment Reminders Among Outpatients in a Universal Health System: Cross-Sectional Study
Source: J Med Internet Res. 2026 Apr 7;28:e81324. doi: 10.2196/81324 (PMC13055944; doi:10.2196/81324)
Supplement: Multimedia Appendix 1 [file jmir-v28-e81324-s001.docx]

**Multimedia**

Appendix 1. The Six SMS Message Types Designed and Developed in This Study (English-Chinese Version)

| Type of SMS TXT Reminder | Wording of SMS Content  in this study | 簡訊提醒  類型 | 本研究設計之簡訊內容  文字措辭結構 |
| --- | --- | --- | --- |
| General Reminders | Hi! You have an appt. with [Dr Name] on the [Morning/Afternoon/ Evening] of [Date]. To cancel or reschedule, please visit [clinic or hospital] booking system. We care about your health! | 一般簡訊 | 您好，提醒您在[00月00日][上午/下午/晚上]與[醫師姓名]有約。若要取消或變 更，請至[醫院或診所名稱]預約掛號系統更改。我們關心您的健康! |
| Prior MA Behavior | Hi! You have an appt. with [Dr Name] on the [Morning/Afternoon/ Evening] of [Date]. Your Appt. No. is [Appt. No.]. If this MA results in a total of three no-shows without prior cancellation, you will no longer be eligible for remote booking. Future appt. must be made in person at the [clinic or hospital]. To cancel or change, please visit [clinic or hospital] booking system. We care about your health! | 先前失約紀錄簡訊 | 您好，提醒您在[00月00日][上午/下午/晚上]與[醫師姓名]有約，看診號是[00]號。如您沒有前來 看診或未取消預約累計三次，下次看診只能到現場掛號。若要取消或變更，請至[醫院或診所名稱]預約掛號系統更改。我們關心您的健康。 |
| Joint Empathy Communication  Type Ⅰ: Provider-Focused | Hi! You have an appt. with [Dr Name] on the [Morning/Afternoon/ Evening] of [Date]. Your Appt. No. is [Appt. No.]. If you don’t show up and don’t cancel, it may cause trouble for medical staff. To cancel or reschedule, please visit [clinic or hospital] booking system. We care about your health! | 同理心簡訊  (類型Ⅰ: 以醫療服務提供者為焦點) | 您好，提醒您在[00月00日][上午/下午/晚上]與[醫師姓名]有約有約，您是掛[00]號。如您沒有前來看 診並沒有取消，將會造成醫護人員的麻煩，若要取消或變更時段，請至[醫院或診所名稱]預約掛號系統更改，我們關心您的健康。 |
| Joint Empathy Communication  Type Ⅱ: Patient-Focused | Hi! You have an appt. with [Dr Name] on the [Morning/Afternoon/ Evening] of [Date]. Your Appt. No. is [Number]. If you miss your appt. without cancellation, it may affect other patients' access to care. To cancel or reschedule, please visit [clinic or hospital booking system]. We care about your health! | 同理心簡訊  (類型Ⅱ: 以影響其他病人權益為焦點) | 您好，提醒您在[00月00日][上午/下午/晚上]與[醫師姓名]有約，您是掛[00]號。如您沒有前來看診並沒有取消，將會影響他人權益，若要取消或變更時段，請至[醫院或診所名稱]預約掛號系統更改，我們關心您的健康。 |
| Gentle & Friendly  (Physician-Follow-Up) | Hi! [Dr Name] cares about you. You have a [specialty] appt. on the [Morning/Afternoon/ Evening] of [Date]. To cancel or reschedule, please visit [clinic or hospital] booking system. We care about your health! | 醫師關懷簡訊  (非首次就診) | 您好，[醫師姓名]關心您，提醒您在[00月00日][上午/下午/晚上]有預約[科別名稱]門 診。若要取消或變更時段，請至[醫院或診所名稱]預約掛號系統更改，我們關心您的健康。 |
| Supportive Reminder | Hope you're doing well! [Dr Name] cares about your health. You have a [specialty] appt. on the [Morning/Afternoon/ Evening] of [Date]. Your Appt. No. is [number]. To cancel or reschedule, please visit [clinic or hospital] booking system. We care about your health! | 支持性簡訊  (以日常問候方式) | 最近好嗎? [醫師姓名]關心您的身體健康，提醒您在[00月00日][上午/下午/晚上]有預約[科別名稱]門診，您是掛[00]號。若要取消或變更時段，請至[醫院或診所名稱]預約掛號系統更改，我們關心您的健康。 |

Note: (1) MA= Miss Appointment; (2) Appt.= Appointment; (3) Appt. No.=Appointment Number;
